# Criticism on “Two Cases of Double Spontaneous Dislocation of the Lens”

**Published:** 1870-02

**Authors:** B. Joy Jeffries

**Affiliations:** 15 Chestnut Street


### Criticism on "Two Cases of Double Spontaneous Dislocation of the Lens."

These cases are reported by Dr. H. W. Williams, of Boston, in the number of this Journal, for January 6, 1870. I think the treatment in the first case and the diagnosis in the second are open to criticism.

A man, æt. 35, noticed a quivering of the irides when 18 years old. Eighteen months ago the lens of the right eye was dislocated into the anterior chamber, which accident has repeatedly occurred since, "not generally accompanied with pain." When he applied, the lens had been four days in the anterior chamber, "giving him a dull pain in the globe, and causing very slight injection." Dr. Williams reports the vision of the other eye to be one-third, using concave six; and for reading, "good." We are not told whether the patient was myopic before the quivering of the irides was noticed; *i. e.* whether the myopia was connected with relaxatian or partial rupture of the suspensory ligament of the lens.

Whether the dislocated lens was in its capsule or not is not reported—merely that "it looked like a drop of oil." The lens was got back behind the iris by rubbing the cornea with the lid whilst the patient was on his back in a dark room. "Three large doses of extract of calabar bean were put into the right eye, producing an evident but slight diminution in the size of the pupil." This did not prevent the lens from tumbling forward again "upon making slight movement." "It is not unlikely that some accommodative effort of the eye may have had an agency in the displacement." How this is possible Dr. Williams does not undertake to say. But if it were, why, then, was the patient "dismissed with directions to put a square of calabar gelatine into the eye every hour, *which would cause constant accomodative effort?* Three large doses had not in an hour shut the pupil enough to hold the lens behind the iris. When there, the lens was not in proper position, it must be noticed, but slipping about; therefore, if "a permanent barrier to the escape of the lens into the anterier chamber" *had been* brought about by the calabar bean, the patients vision would not have been good. Moreover, the extract of calabar bean cannot be continuously applied to the eye even as atropine can. Therefore I hold that the advice to continue its use was not good. As it proved, by "the next day the lens had been several times displaced."

Nothing is said in this report as to the present or future necessity of removing the dislocated lens, or as to the patient's being advised in reference to this point. If this was done, I of course have no further comment; but if not, then I hold it was not good surgery to dismiss the patient without operating or advising it. The whole weight of evidence is in favor of always removing a dislocated lens, especially when it cannot be kept behind the iris. Experience shows it is a "foreign body," likely to produce any trouble, from "a dull pain in the globe with slight injection," as in Dr. Williams's case, within four days, up to ulceration and perforation of the cornea, as was seen by Prof. Graefe; that being the best way nature can extract it. In vol. lxxiv., p. 73, of this Journal, Dr. Williams reports extracting a dislocated lens which was opaque. If this one was left because transparent, I simply say I cannot regard it as good surgery. The ophthalmic journals and text books are full of reports of partial or total displacement of the lens, and experience has fully demonstrated that the sooner a dislocated lens is removed the better for the patient. I forbear tedious quotations from Græfe, the London observers and others.

In the second case, "a boy of about 12, both of whose eyes exhibited partial lateral displacement and slight opacity of the crystalline;" I hold the diagnosis should have been, not "spontaneous dislocation," but *congenital malposition*, as has been often observed. Dr. Williams does not state the amount of the displacement, *i. e.*, whether light entered the eye at one side of the lens, and the mental imbecility of the patient prevented his ascertaining his vision.

B. JOY JEFFRIES.

15 Chestnut Street—*Boston Medical Journal.*

